# Percutaneous compression plate versus proximal femoral nail anti-rotation in treating elderly patients with intertrochanteric fractures: a prospective randomized study

**DOI:** 10.1007/s00776-013-0468-0

**Published:** 2013-10-02

**Authors:** Qingshan Guo, Yue Shen, Zhaowen Zong, Yufeng Zhao, Huayu Liu, Xiang Hua, Hui Chen

**Affiliations:** Trauma Center, Institute of Surgery Research, Daping Hospital, Third Military Medical University, No. 10 Yangtze River Road, Yuzhong District, Chongqing, 400042 People’s Republic of China

## Abstract

**Background:**

The treatment and management of hip fracture poses a great challenge for clinicians in osteology and surgery. The aim of this study is to compare the clinical effectiveness of the percutaneous compression plate (PCCP) versus proximal femoral nail anti-rotation (PFNA) in the treatment of intertrochanteric fractures in elderly patients.

**Methods:**

A prospective randomized study was carried out from January 2008 to October 2011 involving 90 elderly patients with intertrochanteric fractures (90 hips) who underwent minimally invasive surgery using the PCCP or PFNA. Evaluation variables, including operation time, intra- and perioperative blood loss, duration of hospital stay, incidence of postoperative complications, and final clinical outcomes by the end of follow-up, were used to compare the benefits of these two implants.

**Results:**

Among 90 subjects, 45 received PCCPs and 45 received PFNAs. The baseline characteristics of the two groups were comparable. The median follow-up time was 16.9 months (12–24 months). In the PCCP group, the mean operative time was 53 min (40–75 min), and the mean intra- and perioperative blood losses were 100.7 ml (60–150 ml) and 916 ml (433–1339 ml), respectively, which were significantly lower than those in the PFNA group. Nevertheless, there was no statistical difference in the incidence of postoperative complications and final clinical outcomes including pain complaints, range of motion of the hip, postoperative hip function at 12 months, and the recovery of walking ability to pre-injury status between these two implants.

**Conclusions:**

Overall, the PCCP and PFNA appear to have similar clinical effects in treating elderly patients with intertrochanteric fractures, although the PCCP provided shorter operation times and less blood loss than PFNA. Both implants discussed were demonstrated to be ideal for the treatment of femoral intertrochanteric fractures in elderly patients.

## Introduction

Fracture of the proximal femur, generally termed “hip fracture,” is one of the most common and severe fractures occurring in the elderly population. It has been reported that 90 % of hip fractures occur in patients over the age of 65 [1]. When compared with other fractures in this population, hip fracture has greater associated rates of death and disability as well as higher medical expenses [1, 2]. During the last 25 years, the incidence of hip fracture has increased rapidly, and it is estimated that 7.3–21.3 million individuals will suffer from this injury globally in 2050 [3, 4]. Therefore, the treatment and management of hip fracture pose great challenges for clinicians in osteology and surgery.

The primary goal for the treatment of intertrochanteric hip fracture is to achieve minimal mortality and morbidity, low re-operation rates, and early successful run-up to sustainable mobility. The basic strategy for achieving this goal greatly depends on the quality of fracture fixation, including biomechanical stability and rigidity [5, 6]. Currently, the sliding hip screw is the most widely used implant for fixation of intertrochanteric hip fracture and thus serves as a benchmark in this field [7]. In elderly patients, however, this surgical procedure is always associated with substantial intra- and perioperative blood loss and severe soft-tissue damage [8, 9]. Therefore, minimally invasive surgical techniques are being developed in order to overcome these problems implicit in sliding-screw fixations [9]. The percutaneous compression plate (PCCP) and proximal femoral nail anti-rotation (PFNA) are recently developed devices designed for minimally invasive surgery in the treatment of hip fractures, and they have been widely used in elderly patients with demonstrated clinical effectiveness [10–12]. Researchers have also performed numerous clinical studies to compare either the PCCP or PFNA with other orthopedic implants [13–16]. Nevertheless, reports on the clinical effectiveness of the PCCP versus PFNA in elderly patients with intertrochanteric fractures are quite few.

In order to compare the clinical effects of the PCCP versus PFNA in the treatment of hip fractures in elderly patients, we conducted a prospective randomized study from January 2008 to October 2011 involving 90 elderly patients with intertrochanteric fractures who underwent minimally invasive surgery using the PCCP or PFNA. Evaluation variables, including operation time and intra- and perioperative blood loss, incidence of postoperative complications, and final clinical outcomes at the end of follow-up, were used to compare the benefits of these two implants.

## Materials and methods

### Patients

**Fig. 1:**
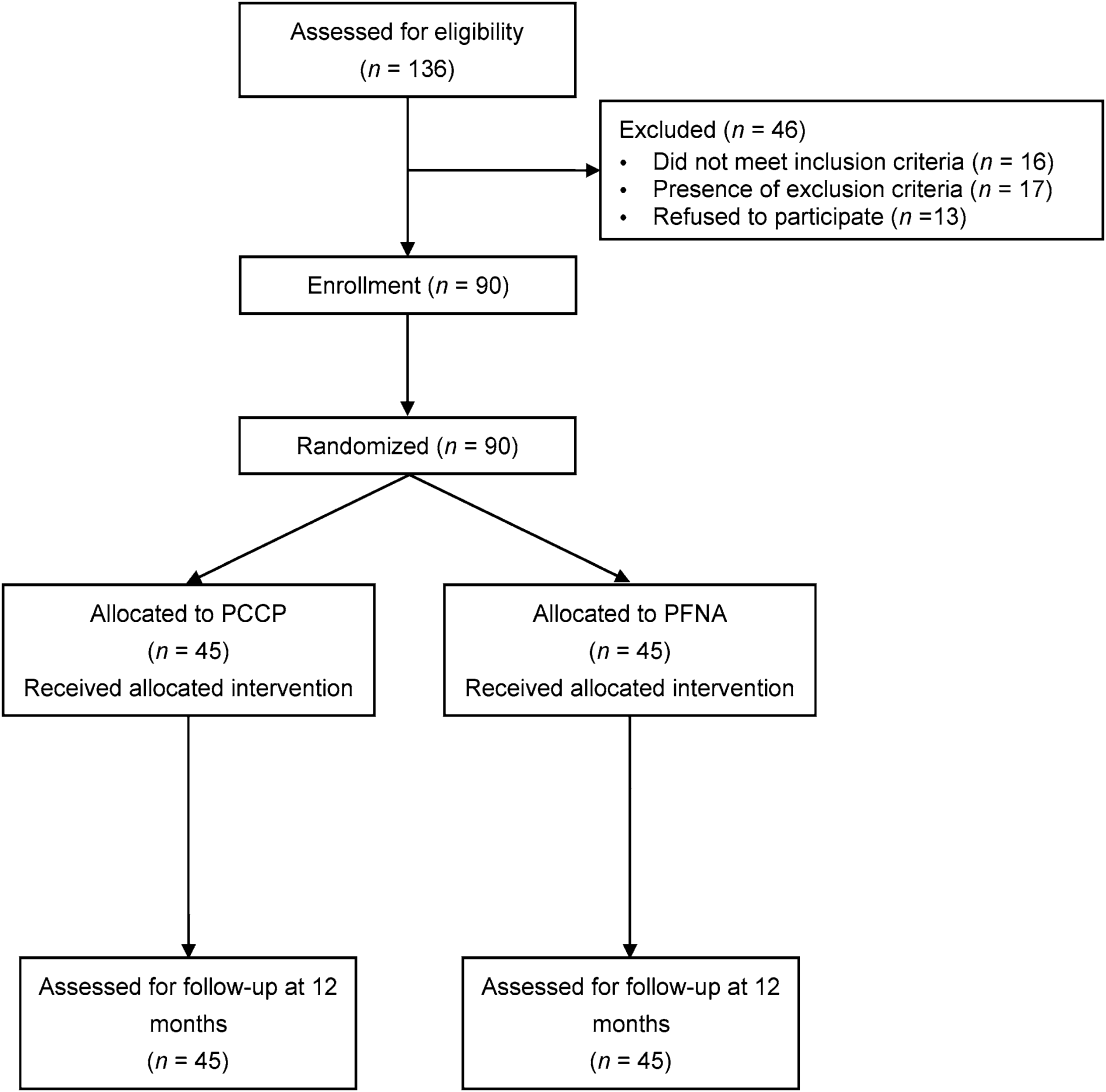
Flow chart of the enrollment of patients for the percutaneous compression plate (PCCP) and proximal femoral nail anti-rotation (PFNA) groups

This study was approved by the Ethics Committee of the authors’ institution. The inclusion criteria were: (1) being older than 60 years (≥60 years); (2) having intertrochanteric fractures of type 31A1 and 31A2 based on the Orthopedic Trauma Association (OTA) classification; (3) an American Society of Anesthesiologists (ASA) Score of I-IV. The exclusion criteria were: (1) younger than 60 years (<60 years); (2) subtrochanteric fractures (type 31A3 in the OTA classification); (3) an ASA score of V; (4) existing or previous fractures in the same or contralateral hip; (5) injuries that could affect the outcome measures; (6) abnormalities that could affect the outcome measures. A total of 136 patients were assessed for eligibility between January 2008 and October 2009. Among them, 33 patients were excluded on the basis of inclusion and exclusion criteria, and 13 refused to participate. Finally, 90 patients (90 hips) were enrolled in this study (Fig. 1). Written informed consent was obtained from each patient or the family members if the patients were incapable of consent.

The patients were randomized into two groups,the PCCP (*n* = 45) or PFNA (*n* = 45), using a sealed-envelope system. The baseline characteristics, including age, gender, cause of fracture, ASA risk score, OTA classification, fracture type based on the Evans-Jensen classification (types I and II as stable and types III–V as unstable), comorbidities, and pre-injury walking ability score (0–9 points according to Parker and Palmer’s method [17]), are described in Table 1.

**Table 1:** The baseline characteristics of enrolled patients

	PCCP	PFNA	Difference of means (95 % CI)	*p* value
Total number of patients	45	45		
Age, years				
Range	63–92	67–89		
Mean (SD)	71.6 (7.5)	74.2 (8.8)	2.6 (−0.83 to 6.0)	0.1350
Gender				0.6657
Male	16	19		
Female	29	26		
Causes of fracture				0.1999
Slip injury	32	24		
Traffic injury	9	15		
Fall injury	4	4		
Others	0	2		
ASA risk score				0.9077
I	6	7		
II	13	12		
III	19	21		
IV	7	5		
OTA fracture classification				0.5248
31A1	18	22		
31A2	27	23		
Fracture type				0.3974
Stable	23	18		
Unstable	22	27		
Comorbidity				
Hypertension and cardiovascular diseases	33	35		
Diabetes mellitus	16	19		
Osteoporosis	5	7		
Sequelae of cerebral infarction	2	2		
Pulmonary infection	2	3		
Chronic renal insufficiency	1	0		
Pre-injury walking ability score				
Range	6–10	6–10		
Mean (SD)	7.4 (2.9)	7.6 (2.3)	0.2 (−0.9 to 1.3)	0.7179

*PCCP* percutaneous compression plate, *PFNA* proximal femoral nail anti-rotation, *SD* standard deviation, *ASA* American Society of Anesthesiologists, *OTA* Orthopedic Trauma Association

### Methods

**Fig. 2:**
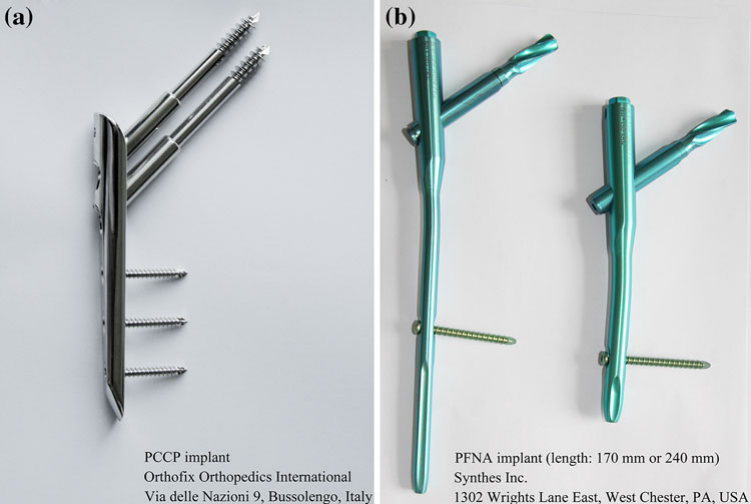
Pictures of the percutaneous compression plate (PCCP) and proximal femoral nail anti-rotation (PFNA) used in this study. **a** The PCCP implant, manufactured by Orthofix Orthopedics International (Via delle Nazioni 9, Bussolengo, Italy); **b** the PFNA implant (length 170 or 240 mm), manufactured by Synthes Inc. (1302 Wrights Lane East, West Chester, PA, USA)

For all patients in both treatment groups, PCCP or PFNA operations were generally performed according to the standard protocols provided by the manufacturer and the procedures described in the previous literature [10, 12, 18, 19]. The PCCP implant (Orthofix Orthopedics International, Bussolengo, Italy) used in this study is composed of a 125-mm plate, two neck screws with lengths from 90 to 140 mm in 10-mm increments, and three shaft screws with lengths from 31 to 43 mm in 3-mm increments (Fig. 2a). The PFNA implant (Synthes Inc., West Chester, PA, USA) was a solid titanium nail with a length of 170 or 240 mm (Fig. 2b). Both the PCCP and PFNA were inserted using a percutaneous technique.

In order to make the operating procedures comparable between the two groups, all operations were performed by expert surgeons who had equal levels of experience with both the PCCP and PFNA. Regional anesthesia was used for both groups. Preoperative antibiotics were administered intravenously to the patients in order to reduce the risk of postoperative infections. All patients underwent implantation on a traction table in a supine position. Blood pressure, pulse, respiration, body temperature, and blood oxygen saturation were monitored during the operation. The operative time was recorded from the start of the skin incision to the time that skin closure was performed by a nurse. Intraoperative blood loss was measured by collection of the suction volume and change in the weight (wet vs. dry) of the sponges. No drains were used. Perioperative blood loss was calculated based on the hemoglobin level and the estimated blood volume of the patient, using the method described by Foss and Kehlet [20]. Estimated blood volume was determined according to gender, body weight, and height [21].

On the first day after surgery, plain anteroposterior (AP) and lateral radiographs were taken to evaluate the reduction of fracture and the position of the PCCP or PFNA implants. All patients were administered prophylactic antibiotics for 3 days. Under the guidance of surgeons, all patients were encouraged to exercise their hip, knee, and ankle joints from the first day post-surgery. They also started to walk with full weight-bearing with a walking aid as soon as possible. For all patients, rivaroxaban (Xarelto, 10 mg/day, p.o.) was administered for 5 weeks after surgery.

Follow-up was conducted in all patients at 3, 6, 9, and 12 months postoperatively and yearly thereafter. Plain AP and lateral radiographs, complications, symptomatic complaints about hip and thigh pain, the range of motion of the hip, Oxford hip score (OHS), and Harris hip score (HHS) at 12 months post-surgery [22, 23] as well as the walking ability score were recorded.

### Statistical analysis

The sample size of this study was calculated based on the OHS at 12 months postoperatively because the OHS at 12 months post-surgery is an important and validated variable in determining clinical outcomes [24]. The calculation was performed according to the results of a pilot study. In this pilot study, the means and standard deviations (SD) of the OHS at 12 months post-surgery in PCCP and PFNA groups was 23.1 ± 3.8 and 25.6 ± 4.3, respectively. Our hypothesis was that there would be a significant difference in the OHS at 12 months post-surgery between the two groups. This requires at least 34 subjects per group with 80 % power (1 − *β*) at the 0.1 significance level (*α*) for statistical analysis, which was calculated using PASS 2008 software (NCSS LLC, Kaysville, UT, USA). Assuming an approximate dropout rate of 20 %, at least 41 subjects were needed for each group. In order to avoid under-powering due to an incorrect estimate of (1 − *β*) and *α*, we decided to recruit 45 subjects per group in this study.

The statistical analysis was performed with SPSS 12.0 software (SPSS Inc, Chicago, IL, USA). All quantitative variables were tested for normality distribution using the Kolmogorov-Smirnov test and presented as mean (SD). Statistical significance of quantitative variables between groups was assessed by Student’s *t* test for independent samples. Statistical significance of categorical variables was assessed by the chi-square test or Fisher's exact test. A value of *p* < 0.05 was considered significant (two-tailed).

## Results

The baseline data of patients, including age, gender distribution, ASA score, and fracture types, were comparable between the two treatment groups. According to the Evans and Jensen classification, unstable fractures occurred in 49 hips and stable fractures occurred in 41 hips. In addition, the majority of patients in both groups had comorbidity with cardiovascular or metabolic disorders (Table 1).

### Intra- and perioperative clinical data

The intra- and perioperative data of the PCCP and PFNA groups are shown in Table 2. All operations were performed by experienced surgeons in our department. The mean PFNA surgery duration was approximately 70 min, which was notably longer than that of PCCP (*p* < 0.0001). Additionally, the intraoperative and calculated perioperative blood loss of patients between the two groups was also significantly different (*p* < 0.0001). Patients receiving PFNA had more blood loss than those receiving PCCP (138.2 vs. 100.7 ml in the mean intraoperative blood loss and 1111 vs. 916 ml in the mean perioperative blood loss, respectively). For the duration of the hospital stay, there was no statistical difference between the PCCP and PFNA groups (7.4 vs. 8.2 days in mean hospital stay) (*p* = 0.3412).

**Table 2:** Intra- and perioperative clinical data

	PCCP	PFNA	Difference of means (95 % CI)	*p* value
Operation time (min)				
Range	40–75	43–116		
Mean (SD)	53.0 (9.4)	66.5 (18.1)	13.5 (7.4 to 19.6)	<0.0001
Intraoperative blood loss (ml)				
Range	60–150	65–250		
Mean (SD)	100.7 (23.5)	138.2 (51.8)	37.5 (20.4 to 54.6)	<0.0001
Calculated perioperative blood loss (ml)				
Range	433–1339	634–1651		
Mean (SD)	916 (44)	1111 (42)	195 (177–213)	<0.0001
Hospital stay (days)				
Range	6–14	5–19		
Mean (SD)	7.4 (3.6)	8.2 (4.3)	0.8 (−0.86 to 2.5)	0.3412

*PCCP* percutaneous compression plate, *PFNA* proximal femoral nail anti-rotation, *SD* standard deviation, *CI* confidence interval

### Postoperative complications by the end of follow-up

Follow-up was obtained from all patients with a median follow-up time of 16.9 months (12–24 months). No patient was lost to follow-up, and no deaths occurred in either group. Data regarding postoperative complications by the end of follow-up are listed in Table 3. Statistical analysis demonstrated that there was no significant difference in postoperative complications between the PCCP and PFNA groups. However, it should be noted that one patient in the PFNA group suffered a femoral shaft fracture, which may have been due to stress concentration. This case was carefully treated with conservative treatment, and full weight-bearing was delayed for 6–8 weeks. After 12 months, the femoral shaft fracture in this patient was healing very well. Additionally, two patients in the PFNA group suffered from fat embolism syndrome (FES). Both patients developed the associated clinical manifestations, including tachypnea, dyspnea, drowsiness, and a nonpappable petechial rash in the chest, axilla, conjunctiva, and neck on the second day after surgery. After receiving high-flow oxygen inhalation as well as albumin and steroid therapy, these clinical manifestations gradually faded.

**Table 3:** Postoperative complications for the PCCP and PFNA groups

Complications	PCCP	PFNA	*p* value
General complications			
Cardiac failure	2	1	0.6077
Pneumonia	1	1	1.0000
Cerebral infarction	2	3	1.0000
Urinary tract infection	1	0	0.4828
Deep venous thrombosis	2	1	0.6077
Urosepsis	1	0	0.4828
Local complications			
Femoral shaft fracture	0	1	1.000
Hematoma	0	1	1.0000
Fat embolism syndrome	0	2	0.4948
Superficial wound infection	1	0	0.4828

*PCCP* percutaneous compression plate, *PFNA* proximal femoral nail anti-rotation

### Clinical outcomes by the end of follow-up

Clinical outcomes of patients in both treatment groups are presented in Table 4. No complications related to fracture union occurred during the follow-up period. All patients achieved clinical and radiological union by the end of follow-up. Symptomatic complaints included hip and thigh pain in 22 patients from the PCCP and PFNA groups during the follow-up period. Nevertheless, statistical analysis revealed that there was no difference in the rate of pain complaints between these two groups. Hip flexion was also similar among the patients treated with the two different implants. Additionally, the mean OHSs were 22.8 and 24.0 for the PCCP and PFNA group, respectively. For HHS, “excellent” and “good” results were achieved in 77.8 % of patients in the PCCP group (35/45) and 82.2 % in the PFNA group (37/45) at 12 months postoperatively. These results indicate that lost hip function was regained in most patients within the first year after implant surgery. Walking ability was also improved to pre-injury status in 58.9 % of all patients in the treatment groups (26 in the PCCP group and 27 in the PFNA group, respectively). However, no statistical significance was observed between these two groups in terms of OHS, HHS, and walking ability score.

**Table 4:** Clinical outcomes by the end of follow-up

	PCCP	PFNA	Difference of means (95 % CI)	*p* value
Hip pain	5	7		0.7578
Thigh pain	4	6		0.7391
Hip flexion				
Range	71–132	69–131		
Mean (SD)	96.1 (15.1)	97.5 (15.0)	1.4 (−5.0 to 7.8)	0.6657
OHS at 12 months postoperatively				
Range	12–35	12–37		
Mean (SD)	22.8 ± 7.0	24.0 ± 7.2	1.2 (−1.8 to 4.2)	0.4249
HHS at 12 months postoperatively				
Excellent/good/fair/poor	24/11/10/0	23/14/8/0		0.7395
Range	70–100	67–100		
Mean (SD)	88.4 (9.0)	87.6 (8.4)	−0.8 (−4.4 to 2.8)	0.6640
Walking ability score				
Range	5–10	5–10		
Mean (SD)	6.9 (1.5)	6.7 (2.8)	−0.2 (−1.1 to 0.74)	0.6738
Recovery of walking ability to pre-injury status				1.0000
Yes	26	27		
No	19	18		

*PCCP* percutaneous compression plate, *PFNA* proximal femoral nail anti-rotation, *SD* standard deviation, *CI* confidence interval, *OHS* Oxford hip score, *HHS* Harris hip score

### Typical cases

#### Case 1

**Fig. 3:**
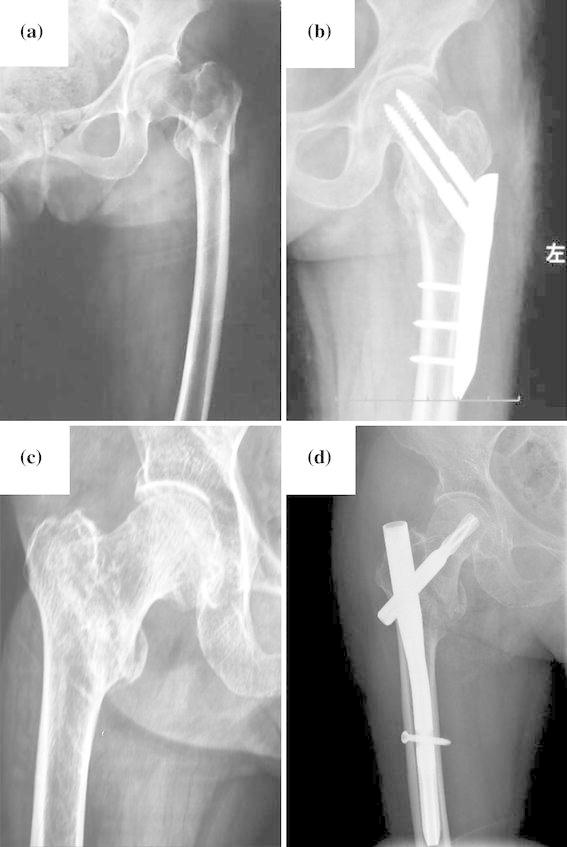
Pre- and postoperative radiographs of representative cases. **a**, **b** A case in the percutaneous compression plate (PCCP) group: **b** reduction and fixation of the fracture were achieved. **c**, **d** A case in the proximal femoral nail anti-rotation (PFNA) group; **d** the fracture was reduced after receiving a PFNA

A 92-year-old female patient was diagnosed with a left femur intertrochanteric fracture (31-A2.3) (Fig. 3a). The comorbidities were severe osteoporosis, coronary arteriosclerotic heart disease (chronic myocardial ischemia and frequent premature ventricular contractions), stage III hypertension, and stage IV chronic kidney disease. She underwent a PCCP operation lasting 55 min and experienced 80 ml intraoperative blood loss. After surgery, the X-ray radiograph showed that anatomic reduction of the fracture had been achieved through appropriate positioning of the plate (Fig. 3b). One week after surgery, this patient was able to walk with a walking aid. Function of the hip joint was greatly improved at 12 months postoperatively with an OHS of 19 and an “excellent” HHS of 91.

#### Case 2

A 75-year-old female patient was diagnosed with a right femoral intertrochanteric fracture (31-A2.2) (Fig. 3c). She had comorbidity with diabetes mellitus and stage III hypertension. PFNA surgery was performed over a 70-min period, and intraoperative blood loss was 110 ml. After surgery, the X-ray radiograph showed the near-anatomic reduction of the fracture had been achieved (Fig. 3d). Postoperative joint function was restored with an OHS of 17 and an “excellent” HHS of 95 at 12 months postoperatively.

## Discussion

The treatment of femoral intertrochanteric fracture in elderly patients remains challenging because these patients always have severe comorbidities, resulting in long hospitalizations and multiple postoperative complications with a high mortality rate [18, 19]. Therefore, internal fixation is usually recommended in the clinic in order to reduce mortality as well as the incidence of coxa vara and limb shortening [25]. Traditional internal fixation has many disadvantages, such as a large wound, heavy blood loss, severe pain, a high incidence of postoperative complications, and slow functional recovery [26, 27]. Minimally invasive surgical techniques for hip fracture are able to overcome these drawbacks. The PCCP and PFNA, the minimally invasive implants most frequently used for internal fixation in current traumatic orthopedics, have been widely accepted for use in the treatment of femoral intertrochanteric fracture in elderly cases [8, 9]. To our knowledge, however, there has been no literature published regarding the comparison between these two implants. In this study, the results showed that there was no obvious difference in the clinical outcomes of patients receiving PCCP and PFNA, except for the surgery duration and intra- and perioperative blood loss.

A percutaneous compression plate was first reported for clinical application by Gotfried in 2000 [18]. This implant is a device with a double-axis and two parallel femoral neck screws, which can withstand high rotational force and provide rotational stability. The small diameter of the screw protects the lateral cortex, thus effectively preventing fracture displacement and allowing immediate full weight-bearing [28]. Comparatively, PFNA has a special helical blade design, which is developed on the basis of the proximal femoral nail (PFN). This special blade has a large surface and increasing core diameter, which guarantees maximum compaction and optimal hold in the bone. Increased rotational and angular stability caused by bone compaction around the PFNA blade can effectively avoid rotation and varus collapse, which has been biomechanically proven [29]. Therefore, generally, both the PCCP and PFNA have desirable mechanical properties for internal fixation of hip fractures. However, in regards to surgical duration and intra- and perioperative blood loss, our results showed there was a significant difference between these two implants. Patients in the PFNA group underwent longer operation times and lost more blood during surgery compared to the PCCP group. This might be attributed to the procedures necessary for femur opening and insertion of the PFNA implant into the medullary canal, which requires much care and time to succeed in correctly positioning the implant in the medullary canal.

Our clinical outcome results showed that postoperative complications were well controlled in both groups and that there was no significant difference in the incidence of postoperative complications between PCCP and PFNA. Furthermore, we did not observe any complications related to fracture union during the follow-up period, which can perhaps be attributed to the extensive experience of the surgeons involved in this study as well as strict postoperative patient management. These findings are also consistent with the results reported in previous studies [16, 30], which report a very low incidence of complications related to fracture union in patients receiving the PCCP or PFNA. Nevertheless, one patient with the PFNA did encounter femoral shaft fracture at the tip of the implant. This complication has been reported to be common when using intramedullary nails for treating proximal femoral fractures. Leung et al. [31] reported a geometric distinction in Gamma nails used in Chinese patients because elderly Chinese have relatively shorter femurs and excessive anterior bowing as compared to American and European patients. The case of femoral shaft fracture in our study also involved a short femur. When the PFNA implant was inserted, the stem likely did not fit the patient’s femur well, thus causing the malposition of the implant in the medullary canal. Additionally, a wedge effect may occur during the introduction process involving the use of a hammer. The malpositioning of the PFNA and the wedge effect produced a stress concentration, thus resulting in the occurrence of femoral shaft fracture in this case.

It should be noted that two patients in the PFNA group developed FES right after surgery. The pathophysiology of FES remains unclear, although two theories, mechanical and biochemical, currently exist, which postulate its occurrence [32]. Previous literature has pointed out that FES is commonly associated with traumatic fracture of the femur, pelvis, or tibia [33]. In addition, there may be a casual correlation between FES onset and intramedullary nailing, and pelvic and knee arthroplasty, although there is much controversy surrounding this issue [32]. In the current study, FES only occurred in the patients receiving PFNA, indicating intramedullary fixation with PFNA might be responsible for its onset. However, further study with a large population is needed to verify this assumption.

Researchers have reported that hip and thigh pain is common, with treatment involving intramedullary fixation [34]. However, our results show that the incidence of hip and thigh pain was relatively lower in both groups when compared with the results reported in previous literature [34]. As to the recovery of hip function and walking ability, there was no significant difference between these two devices. Between both groups, walking ability was recovered to pre-injury status in 58.9 % of patients, which is close to or even higher than the results reported in other literature [34, 35]. This finding indicates that the selection of the PCCP or PFNA as the implant for fixation is not a key determinant of clinical outcomes. General conditions of patients, postoperative exercises, and the multidisciplinary management of preoperative comorbidities and postoperative complications may determine the final outcomes of patients.

Numerous studies have been performed to compare the clinical effect and safety of the PCCP with the dynamic hip screw (DHS), which has been considered as the gold standard treatment for intertrochanteric fracture [26]. As a minimally invasive implant, the PCCP has obvious advantages in regards to blood loss, need for transfusions, and systematic complications, although it was shown to be similar to the DHS in mechanical stability and clinical effect [8, 9]. Furthermore, increasingly surgeons are not considering the PFNA a truly minimally invasive technique because of the potential risk of femoral shaft fracture, more severe tissue damage, and greater blood loss [6]. According to our experience, although the PFNA has the aforementioned disadvantages, they can be easily controlled. Additionally, this device has a broader range of applications than PCCP. For example, it can be used for treating 31A3 fractures, which is a contraindication for the PCCP. More importantly, insertion of the PFNA is able to compact the cancellous bone, providing additional anchoring, which is especially suitable for patients with osteoporotic bone.

In summary, based on our findings, the PCCP and PFNA appeared to have similar clinical effects in treating elderly patients with intertrochanteric fractures. The PCCP was shown to require shorter operation times and less blood loss than the PFNA. However, both were demonstrated to be ideal implants for the treatment of femoral intertrochanteric fractures, especially those that occur in elderly patients with severe pre-existing diseases.
